# Diagnoses behind patients with hard-to-classify tremor and normal DaT-SPECT: a clinical follow up study

**DOI:** 10.3389/fnagi.2014.00056

**Published:** 2014-04-01

**Authors:** Manuel Menéndez-González, Francisco Tavares, Nahla Zeidan, José M. Salas-Pacheco, Oscar Arias-Carrión

**Affiliations:** ^1^Neurology Unit, Hospital Álvarez-BuyllaMieres, Spain; ^2^Morphology and Cellular Biology Department, Universidad de OviedoOviedo, Spain; ^3^Instituto de Neurociencias, Universidad de OviedoOviedo, Spain; ^4^Family Medicine, Hospital Álvarez-BuyllaMieres, Spain; ^5^Nuclear Medicine, Hospital Universitario Central de AsturiasOviedo, Spain; ^6^Instituto de Investigación Científica, Universidad Juárez del Estado de DurangoDurango, Mexico; ^7^Transcranial Magnetic Stimulation Unit, Sleep and Movement Disorders Clinic, Hospital General Dr. Manuel Gea GonzálezMexico City, Mexico

**Keywords:** DaTSPECT, tremor, Parkinson disease, Movement Disorders, SWEDD

## Abstract

The [^123^I]ioflupane—a dopamine transporter radioligand—SPECT (DaT-SPECT) has proven to be useful in the differential diagnosis of tremor. Here, we investigate the diagnoses behind patients with hard-to-classify tremor and normal DaT-SPECT. Therefore, 30 patients with tremor and normal DaT-SPECT were followed up for 2 years. In 18 cases we were able to make a diagnosis. The residual 12 patients underwent a second DaT-SPECT, were then followed for additional 12 months and thereafter the diagnosis was reconsidered again. The final diagnoses included cases of essential tremor, dystonic tremor, multisystem atrophy, vascular parkinsonism, progressive supranuclear palsy, corticobasal degeneration, fragile X–associated tremor ataxia syndrome, psychogenic parkinsonism, iatrogenic parkinsonism and Parkinson's disease. However, for 6 patients the diagnosis remained uncertain. Larger series are needed to better establish the relative frequency of the different conditions behind these cases.

## Introduction

Tremor is an involuntary, rhythmic, oscillatory movement of a body part. It is the most common movement disorder encountered in clinical practice. There is no diagnostic standard to distinguish among common types of tremor, which can make the evaluation challenging. History and physical examination can provide a great deal of certainty in diagnosis. However, some cases showing more than one type of tremor or associating other signs and symptoms are specially difficult. Establishing the underlying cause is important because prognosis and specific treatment plans vary considerably.

PD is a common neurodegenerative disorder characterized by progressive degeneration of dopaminergic neurons in the substantia nigra, with loss of their nerve terminals in the basal ganglia structures, especially in the striatum. The dopaminergic system is the most studied neurochemical system in patients with PD because damage to nigrostriatal neurons is the most important component in the pathophysiology of PD. Clinically it is characterized by the so called “parkinsonian syndrome,” consisting of extrapiramidal signs, including bradykinesia and at least one of the following: muscular rigidity, 4–6 Hz rest tremor and postural instability not caused by primary visual, vestibular, cerebellar, or proprioceptive dysfunction. More than 70 percent of patients with Parkinson disease have tremor as the presenting feature. The classic parkinsonian tremor begins as a low-frequency, pill-rolling motion of the fingers, progressing to forearm pronation/supination and elbow flexion/extension. It is typically asymmetric, occurs at rest, and becomes less prominent with voluntary movement. Although rest tremor is one of the diagnostic criteria for Parkinson disease, most patients exhibit a combination of action and rest tremors (Rodriguez-Oroz et al., 2009). The term “parkinsonisms” refers to a group of neurological disorders characterized by a parkinsonian syndrome. In many cases, the differential diagnosis between Parkinson disease and other neurodegenerative parkinsonisms, or other conditions such as essential tremor, psycogenic, or drug-induced parkinsonism is sometimes difficult. This requires an experienced clinician and time to establish the pattern of progression and response to treatment.

Currently, the diagnosis of tremor remains primarily clinical, but complementary tests may be useful to support the diagnostic process for particularly difficult cases, specially in those where tremor associates with parkinsonian syndrome. Structural imaging, such as computed tomography or magnetic resonance imaging (MRI), is of limited value for differentiating parkinsonian syndromes since structural changes are often only evident by the time the disease is far advanced. CT and MRI neuroimaging do play an important role in the diagnosis of patients with vascular parkinsonism. Radiotracer neuroimaging techniques allow to study the integrity of the dopaminergic nigrostriatal system and are therefore a valuable tool to diagnose neurodegenerative parkinsonisms (Lorenzo Bosquet et al., 2004; Tolosa et al., 2007). Positron emission tomography techniques demonstrate the disruption of selective patterns of regional cerebral metabolism and neurotransmitter systems associated with subcortical degenerations, such as Parkinson's disease, striatonigral degeneration, progressive supranuclear palsy, and corticobasal degeneration (Antonini and Isaias, 2009; Huang et al., 2013). In addition, it allows to determine, where underlying Parkinson's disease may be suspected and whether nigral dysfunction is present in patients with isolated tremor or drug-associated rigidity. However, PET scan is an expensive technique that is not available in most clinical centers. A DaT-SPECT (Dopamine Transporter -DaT- single-photon emission computed tomography), is a less expensive and more widely available technique compared to PET and has already been incorporated into clinical practice. The active ingredient of [123I]FP-CIT SPECT is a cocaine analog, 123I-labeled N-u-fluoropropyl 2b-carbomethoxy-3b-(4-iodophenyl) nortropane ([123I]ioflupane). It binds with high affinity to striatal presynaptic DAT in animals and in humans and helps visualize these neurons with SPECT brain imaging. [123I]FP-CIT SPECT has the advantage of faster kinetics, which allows imaging 3–6 h after injection. DAT is located on the plasma membrane of nerve terminals in a small number of neurons in the brain, especially in the striatum and nucleus accumbens, and in the globus pallidus, cingulate cortex, olfactory tubercle, amygdala, and midbrain. DAT regulates the dopamine concentration in the synaptic cleft through reuptake of dopamine into presynaptic neurons and thus plays a central role in the buffering of the released dopamine (Surasi et al., 2013).

**Figure 1:**
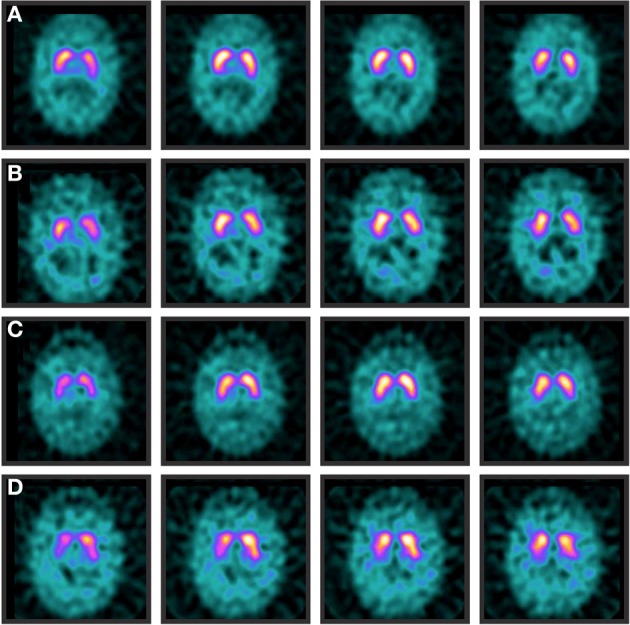
**Normal and abnormal DAT-SPECTs**. Normal DaT-SPECTs of a patient with essential tremor at baseline **(A)** and 42 months later **(B)**. Normal DaT-SPECT of a patient with Parkinson's disease at baseline **(C)**. However, 84 months later **(D)** the scan was abnormal due to a decrease in postsynaptic uptake on the right striatum.

The Food and Drug Administration (FDA) has recently approved the use of DaT-SPECT “to assist in the evaluation of adult patients with suspected parkinsonian syndromes” (Figure 1). Paradoxically, however, adequate neuropathologic validation of Parkinson's disease diagnosis based on DaT-SPECT findings is still lacking and the value of DaT-SPECT for clinical decision-making remains unclear. Generally, the clinical based diagnostic accuracy of Parkinson's disease is mathematically identical to the diagnostic accuracy of DaT-SPECT imaging (6). In terms of differential diagnoses, DaT-SPECT imaging cannot distinguish reliably between Parkinson's disease and other degenerative parkinsonisms, such as multiple system atrophy or progressive supranuclear palsy, whenever evaluated on a case-by-case basis. This important point is specifically recognized in the FDA briefing document (De La Fuente-Fernandez, 2012). Finally, the alternative diagnoses to Parkinson's disease when a patient with hard-to-classify tremor shows a normal (negative) study are not well known yet. Herein, we report a series of patients with different types of tremor or tremor plus parkinsonism, where the clinician decided to perform a DaT-SPECT due to diagnostic difficulties and the DaT-SPECT resulted normal. These patients were followed up for 2 years at least, with the aim of revealing the final diagnoses of these patients.

## Methods

Standard protocol approvals, registrations, and patient consents. The study was approved by the Hospital Álvarez-Buylla and informed consent was given by all family members.

### Subjects

We screened patients with tremor, who had performed a DaT-SPECT and resulted normal. The period of time for screening patients was 5 years. Patients with dementia were ruled out, though patients with mild cognitive impairment were allowed for inclusion (Huang et al., 2013).

### Follow up

Patients included were assessed every 6 months for 2 years at least. Neurological examination was performed to all patients in all visits. Other studies, including a second DaT-SPECT, were done in some cases according to the neurologist criteria. At the end of the follow up period we reconsidered diagnoses based on findings in history, examination, response to treatment and complementary studies. Cases in which we were unable to reach a final diagnosis remained labeled as “uncertain diagnosis.”

### Imaging acquisition

Following thyroid iodine uptake blocking with 500 mg of potassium perchlorate, patients underwent intravenous administration of a single dose (148 MBq)in of DaTSCAN (GE Healthcare). SPECT imaging was carried out 3–6 h later with a dual-headed gammacamera (Philips Healthcare) using LEHR collimators. Data were acquired in a 128 × 128 matrix; zoom 2.19; 180° per head; 20 s per view; 128 views; 158 keV; 15% window; filtered back projection and 2-D Butterworth prefilter; power factor 8; cut off 0.6.

### Data analysis

DaTa analysis was acquired visually and semi-quantitatively with Xeleris 2.0 software (GE Healthcare). In order to analyze the dopaminergic deficit, two different methods were performed: visual interpretation and semi- quantification with classical manual ROIs (Region of Interest) method. For visual interpretation, a nuclear medicine physician examined the hardcopy images and classified the SPECT images into two different patterns: normal, showing a symmetrical uptake bilaterally in putamen and caudate nuclei; and abnormal, with different levels of uptake reduction in one or both caudate and/or putamina. The semi- quantitative evaluation method allows to calculate binding ratios by comparing activity in striatum with activity in an area of low DaT concentration (usually the occipital area). Three consecutive slices with the highest striatal image count were selected and summed up to a single slice. The ratio of specific to non-specific binding was calculated by standardized two dimensional ROIs, derived from an anatomical brain atlas, which were placed bilaterally over the striatum with subregions for caudate and putamen. A ROI over the occipital cortex was used as reference region to assess non-specific binding. Specific FP-CIT tracer uptake was calculated for caudate and putamen using the formula: [Striatal binding ratio = mean counts of striatal ROI-mean counts of occipital ROI/ mean counts of occipital ROI].

## Results

### Sample description

We screened 34 patients with tremor who had underwent a DaTSPECT due to diagnostic difficulties and the result of DaTSPECT was negative (normal). Four of the patients screened were ruled out for suffering from dementia. The demographic and main clinical features of the patients included are summarized in Table 1.

**Table 1:** **Demographics and clinical features of the patients included in the study**.

Mean age	67 years (57–79)
Gender distribution	17 males 13 females
Mean age at onset	61 years (52–74)
Evolution time at inclusion	35 months (8–58)
Mean MMSE	26 (20–30)
Mean UPDRS	24 (12–36)
Mean L-DOPA dose	564 mg (0–1200)

### Diagnoses and follow-up

**Figure 2:**
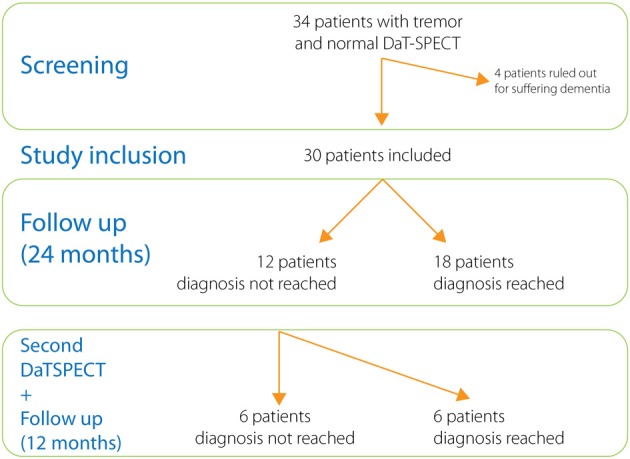
**Study design**.

After a follow up period (24 months) the diagnoses of all cases were reconsidered and recorded (Figure 2). The main clinical features allowing us to reach the diagnoses are included under Table 2. Diagnoses included cases of essential tremor (6 cases), dystonic tremor (2 cases), multisystem atrophy (2 cases), vascular parkinsonism (2 cases), Parkinson's disease (1 case), progressive supranuclear palsy (1 case), corticobasal degeneration (1 case), Fragile X–associated tremor ataxia syndrome (1 case), psychogenic parkinsonism (1 case), and iatrogenic parkinsonism (1 case) (see Table 3). In twelve cases we were unable to reach a diagnosis. These patients were labeled as “uncertain diagnosis.” They underwent a second DaT-SPECT and were followed for 12 months more, with the following results: in 4 patients the second DaT-SPECT resulted abnormal and they were finally diagnosed with Parkinson's disease (see Discussion for details); in 8 patients the second DaT-SPECT resulted normal again—of those 2 were finally diagnosed with dystonic tremor, whereas 6 remained undiagnosed and labeled as “uncertain diagnosis.”

**Table 2:** **Main clinical features of every subject included in the study listed by final diagnoses**.

**Final diagnos**	**Tremor**	**Rigidity**	**Brady kinesia**	**Postural stability**	**Gait**	**Others**	**ResponL-dopa**
ET1	Sym, P,A,V,C	+	−	+	+	MCI	NT
ET2	Sym, P,A,V,C	−	−	+	+		NT
ET3	Asym, P,A	−	−	+	+		−
ET4	Sym, P,A,V,C	+	−	+	+	MCI	NT
ET5	Sym, P,A	−	−	+	+		−
ET6	Sym, P,A,C	−	−	+	+		NT
PD1	Asym, P, R	+, F	+	+	−		++
PD2	Asym, R	+, F	+	+	+	MCI	++
PD3	Asym, R	+, F	+	−	+		++
PD4	Asym, P, R	+, F	+	−	+	MCI	++
PD5	Asym, R	+	+	−	+		++
DT1	Asym, R	−	−	+	+	Dystonia	−
DT2	Asym, R, J	+	−	−	−	Dystonia	−
DT3	Asym, R	−	−	+	+	Dystonia	−
DT4	Asym, R	−	−	+	+	Dystonia	−
MSA1	Sym, P, J	+	+	−	−	UMS AD AC	+
MSA2	Asym, P, A. R, J	+, F	+	−	−	MCI UMS Stridor	+
VP1	Sym, P,A	+	+	+	+	UMS	+
VP2	Sym, P,A	+	−	+	+	UMS	−
PSP1	Sym, P	+, F	+	−	−	SGP AS	−
CBD1	Asym, P, R	+	+	−	−	MCI MC Apraxia	−
FXTAS	Sym, P,A,R,V,C	+	−	−	−	Ataxia	−
Psycho1	Sym, P,A, R	−	−	−	−	Incons Distrac	NT
Iatro1	Sym, P,A, R	+	+	+	+	Dyskinesia	+
?1	Asym, P, R	+	−	+	+	MCI	−
?2	Asym, P, R	−	+	−	−	MCI UMS	−
?3	Asym, P, R	−	+	+	+	MCI MC Apraxia	−
?4	Asym, P, R	+, F	−	−	−	Dystonia	−
?5	Sym, P,A,R,V,C	−	−	−	−	MCI Ataxia	+
?6	Asym, P, R	−	−	+	+	MCI Apraxia	−

*Acronims and clues by columns: Final diagnoses: ET, Essential Tremor; PD, Parkinson's disease; DT, Dystonic tremor; MSA, Multiple system Atrophy; VP, Vascular parkinsonism; PSP, Progressive supranuclear palsy; CBD, Corticobasal degeneration; FXTAS, Fragile X–associated tremor ataxia syndrome; Psychog, Psychogenic parkinsonism; Iatro, Iatrogenic parkinsonism; ?, unclear diagnosis. Tremor: - = Absent Sym, Symmetrical in limbs; Asym, Asymmetrical in limbs; R, Rest tremor; P, Postural tremor; A, Action tremor; J, Jerky tremor; V, Vocal tremor; C, Cephalic tremor. Rigidity: + = present, - = absent, F, Froment sign positive; Bradykinesia: + = present, - = absent, Postural stability: + = normal, - = abnormal, Gait: + = normal, - = abnormal. Others: UMS, upper motor neuron signs; MCI, Mild cognitive imparirment; AD, Autonomic dysfunction; AC, Antecollis; SGP, Supranuclear gaze palsy; AS, Applause sign; MC, Myoclonus; Incons, inconsistent exploratory signs; Distract, Distractability. Response to L-Dopa: ++ = very good, + = good, - = poor or negative, NT, not tested*.

**Table 3:** **Cases diagnosed after the first and second DaT-SPECTs and follow-up periods**.

**Diagnoses**	**DaT-SPECT (prefollow-up)**			**Total (final diagnoses)**
	**After first follow-up period (*n* = 30)**	**After second follow-up period (12/30)**		
	**Normal**	**Normal**	**Abnormal**	
Essential tremor	6	−	−	6
Parkinson's disease	1	−	4	5
Dystonic tremor	2	2	−	4
Multisystem atrophy	2	−	−	2
Vascular parkinsonism	2	−	−	2
Progressive Supranuclear Palsy	1	−	−	1
Corticobasal Degeneration	1	−	−	1
Fragile X–associated tremor ataxia syndrome	1	−	−	1
Psychogenic parkinsonism	1	−	−	1
Iatrogenic parkinsonism	1	−	−	1
Uncertain	12	6	−	6

**Figure 3:**
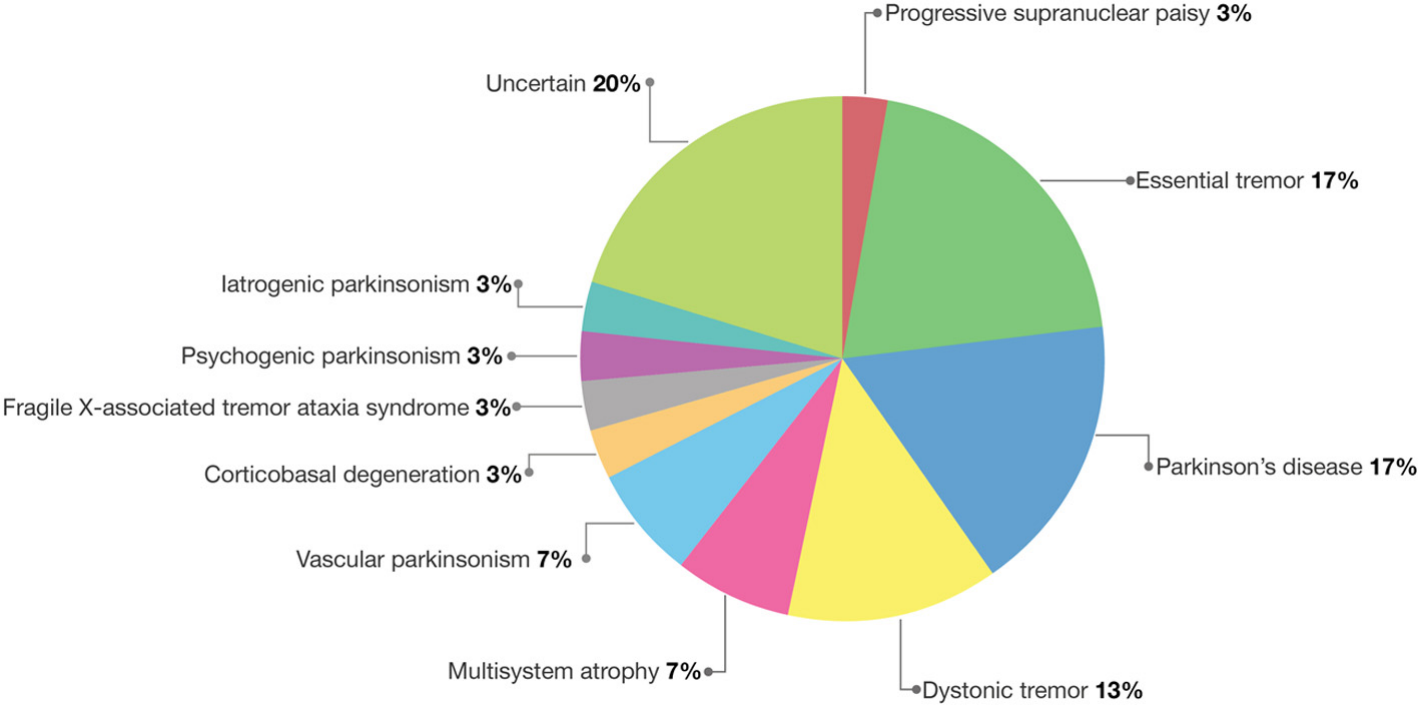
**Pie graph showing the distribution of final diagnoses and relative frequencies**.

Thus, the final diagnoses included cases of essential tremor (6 cases), Parkinson's disease (5 cases), dystonic tremor (4 cases), multisystem atrophy (2 cases), vascular parkinsonism (2 cases), progressive supranuclear palsy (1 case), corticobasal degeneration (1 case), Fragile X–associated tremor ataxia syndrome (1 case), psychogenic parkinsonism (1 case), and iatrogenic parkinsonism (1 case) (Table 3 and Figure 3).

## Discussion

The acronym SWEDDs (scans without evidence of dopaminergic deficits) (Schneider et al., 2007), relatively recent in usage, arose from the clinical trial literature for Parkinson's disease, in which patients were imaged with ^18^F-dopa PET or DaT-SPECT in order to monitor disease progression, revealing that a substantial proportion of clinically diagnosed cases of Parkinson's disease had normal SCANS (4–15%) and were therefore designated as SWEDDs (Schneider et al., 2007; Bajaj et al., 2012). Thus, the term SWEDDs can be leveled at any patient diagnosed at first with Parkinson's disease but subsequent functional imaging assessments do not confirm the presynaptic, dopaminergic deficiency origin.

From the semiological point of view, SWEDDs phenotypes vary in much the same way as Parkinson's disease phenotypes do. There are two broad Parkinson's disease phenotypes, akinetic-rigid (also known as postural instability gait disorder variant -PIGD) and tremor dominant (also known as tremulous Parkinson's disease). In the same way, SWEDDs patients can be subdivided into tremor dominant and non-tremor dominant (or tremor absent) subtypes. With this and the knowledge of the clinical picture of other parkinsonisms in mind, the clinician in front of a patient with SWEDDs can usually reach a diagnosis, in many cases after a follow up period (De La Fuente-Fernandez, 2012). In the following, we discuss in detail the different diagnoses we found and describe the main clues to consider each one.

Although abnormal in most patients with Parkinson's disease, a normal DaT-SPECT is not capable to totally exclude the disease (Vlaar et al., 2007, 2008; Serrano Vicente et al., 2009). A meta-analysis was conducted by Vlaar and colleagues to review the diagnostic accuracy of SPECT to differentiate between early phase of PD and normalcy. All the 6 cross-sectional studies (using presynaptic tracers) with patients with known PD in an early stage (Hoehn and Yahr score of 2 or lesser) had a specificity of 100% and the sensitivity varied from 8% to 100% (Vlaar et al., 2007, 2008). A possible explanation for the low sensitivity found in some studies is that DaT-SPECT can be normal in the very initial stages of the disease. Indeed we diagnosed 1 case with Parkinson's disease in spite of having a normal DaT-SPECT. At least two studies found that in cases that undergo a second DaT-SPECT the accordance of the result with the final clinical diagnosis was higher in the second DaT-SPECT than in the first one (Vlaar et al., 2007, 2008; Serrano Vicente et al., 2009), thus suggesting that the result of the first DaT-SPECT of patients with Parkinson's disease can be negative if performed too early and become positive later on. In our study 4 out of 12, in which a second DaT-SPECT was performed, resulted abnormal. All these patients responded well to L-DOPA or agonists and had a typical parkinsonian syndrome, therefore they were diagnosed with Parkinson's disease eventually. Another explanation for the relatively high number of false-negative Parkinson's disease patients is the quantitative analysis of the SPECT scans. When recalculating the accuracy of DaT-SPECTs to differentiate patients with idiopathic Parkinson's disease from those with essential tremor using visual qualitative judgment instead of quantitative analysis studies report the sensitivity increased from 80% to 94%, negative predictive value from 48 to 71%, specificity and positive predictive value stayed unchanged (Vlaar et al., 2008; Marshall et al., 2009). However, the rule is that the vast majority of patients with Parkinson's disease have an abnormal DaT-SPECT and a normal DaT-SPECT should always make us reconsider the diagnosis of Parkinsons, even in early stages.

Essential tremor may be confused with parkinsonism for several reasons. Firstly, although essential tremor is characterized by a tremor that is exacerbated by posture-holding and action, these patients may also have a tremor at rest (Cohen et al., 2003; Rajput et al., 2004), whereas parkinsonian patients may also have a postural tremor. Secondly, even though the other cardinal features may be discriminating, some essential tremor patients do have mild rigidity (Rajput et al., 2004), whereas some patients with early Parkinson's disease may present with an isolated rest or postural tremor without any other features of parkinsonism. Some patients with a late onset, markedly asymmetrical postural tremor that was diagnosed initially as essential tremor but who went on after many years to develop typical Parkinson's disease (Chaudhuri et al., 2005). When in doubt, follow up and response to treatments are crucial as those with PD will eventually progress to develop clear parkinsonian features and respond to dopaminergic therapies. Multiple studies report that DaT-SPECT can distinguish parkinsonian syndrome from essential tremor (Asenbaum et al., 1998; Tolosa et al., 2007; Antonini and Isaias, 2009; Surasi et al., 2013). In fact, essential tremor was usually chosen as the comparator disorder for many studies since it was thought to have normal striatal DaT. However, DaT-SPECT in essential tremor is not always normal, or at least not as normal as in controls; further suggesting a potential link of some essential tremor cases with Parkinson's disease (Isaias et al., 2008; Antonini and Isaias, 2009; Gerasimou et al., 2012; Labiano-Fontcuberta and Benito-Leon, 2012). We found essential tremor to be the most common cause of tremor with normal DaT-SPECT (6/30 cases).

Primary adult-onset dystonia can present with an asymmetric resting arm tremor, with impaired arm swing and sometimes also facial hypomimia or a jaw tremor, but without evidence of true akinesia (Schneider et al., 2007). Tremor is a relatively common feature occurring in about 17% of patients with primary late-onset dystonia (Defazio et al., 2013). The association between tremor and dystonia spread suggests that this form of tremor may be a dystonic manifestation. Tremor may be classified either as dystonic tremor or tremor associated with dystonia (TAWD) according to the Movement Disorder Society Consensus Statement (Deuschl et al., 1998). Similarities in phenotypic features of dystonic tremor and TAWD predominate over differences, suggesting that the two forms of tremor may be manifestations of the same disease (Defazio et al., 2013; Tinazzi et al., 2013). Differences in gender, body distribution and temporal thresholds of tremor between patients with dystonia and tremor and those of patients with essential tremor also indicate that tremor in dystonia and essential tremor are different entities (Defazio et al., 2013; Tinazzi et al., 2013). Patients with primary adult-onset dystonia show normal DaT-SPECT studies (Schneider et al., 2007). Neurophysiological studies also show that the pattern of plasticity of sensorimotor circuits in patients with tremor dominant SWEDDs resembles the pattern seen in dystonia patients and differs from the pattern found in patients with Parkinson's disease (Schwingenschuh et al., 2010), thus suggesting that many patients with tremulous SWEDDs may have in fact dystonia (Bajaj et al., 2010). In our series 4/30 patients were diagnosed with dystonic tremor. All these patients had a tremoric parkinsonian syndrome with a clear dystonic component in their tremor. Some dystonic signs may also be present in Parkinson's disease, though these are usually mild. In addition, patients with dystonic tremor do not respond to L-DOPA or dopaminergic agonists (Bajaj et al., 2010).

As indicated by its name, progressive supranuclear palsy (PSP) is characterized by a supranuclear gaze palsy with hypometric or slow saccades, particularly on downgaze (Stamelou et al., 2010). However, in the early stages, these abnormalities are often absent and occasionally they do not develop at all (Nath et al., 2003; Williams et al., 2005). DaT-SPECT studies are usually abnormal as in Parkinson's disease. In our series only 1 case was diagnosed with PSP. This case progressed rapidly to the development of the typical picture of PSP with cognitive dysfunction and poor response to L-DOPA.

Fragile X–associated tremor ataxia syndrome (FXTAS) defined by fragile X mental retardation 1 (FMR1) premutation, cerebellar ataxia, intentional tremor, middle cerebellar peduncle hyperintensities in MRI and peripheral neuropathy (Jacquemont et al., 2003; Apartis et al., 2012). About a half of patients with FTAX have abnormal DaT-SPECT (Apartis et al., 2012). One case in our series was diagnosed with FXTAS during follow up based on the genetic study (90–100 CGG Repeats in Gen FMR1). This case was a woman who had been followed in our center for years due to a tremoric parkinsonian syndrome. She was put on several antitremoric treatments, but her tremor only responded to Primidone.

Most cases of symptomatic parkinsonism are vascular parkinsonism. Basal ganglia infarct is a relatively uncommon cause of parkinsonism, but diffuse cerebrovascular disease is much more frequent (Sibon and Tison, 2004; Thanvi et al., 2005). Qualitatively DaT-SPECT images are normal in about a third of patients with vascular parkinsonism. The use of different visual score patterns showed higher ability to differentiate vascular parkinsonism from Parkinson's disease. Semi-quantitative analysis showed significantly higher uptake in the striatum, caudate and putamen in vascular parkinsonism. Among patients with vascular parkinsonism, falls were the only clinical feature that demonstrated a correlation with the SPECT visual pattern (Benitez-Rivero et al., 2013). In spite of the fact that most cases of symptomatic parkinsonism are vascular parkinsonism we only had 2 cases in our series. This is probably due to the fact that in our center all patients with parkinsonian syndrome undergo neuroimaging and those with high vascular load are not asked to perform a DaT-SPECT, for this reason they were not included in this study. However, two patients with vascular risk factors, low vascular load and a normal DaT-SPECT at screening were finally diagnosed with vascular parkinsonism due to fast progression to the typical vascular parkinsonian syndrome and increase in the vascular load in new neuroimaging studies. This suggests that early parkinsonian syndrome may be due to vascular lesions in spite of low vascular load in initial neuroimaging studies, and both clinical and neuroimaging follow up are needed when vascular risk factors are present.

Many drugs can cause parkinsonism, most commonly antipsychotics and antiemetics; and more rarely others such as methyldopa, calcium antagonists, and sodium valproate among many others. Therefore, before making the diagnosis of Parkinson's disease, it is important to check the patient's medication (both current and previous). If the patient is on a relevant drug, it should be stopped if possible, and the patient followed up. Drug induced parkinsonism can take several months to resolve after the drug is discontinued. And even if the symptoms do improve, follow up has shown that a few of these patients will later develop Parkinson's disease, suggesting that the drug had unmasked subclinical Parkinson's disease (Chabolla et al., 1998; Lopez-Sendon et al., 2013). If the drug cannot be stopped, it can be very difficult to distinguish drug induced parkinsonism from idiopathic PD. In this situation, functional imaging with a dopamine transporter ligand may be useful, because patients with pure drug induced parkinsonism have normal scans (Booij et al., 2001; Marshall et al., 2009; Tinazzi et al., 2012). However, contrary to what one would expect, several studies have encountered abnormal DaT-SPECT findings in a surprisingly high number of patients clinically diagnosed as having drug-induced parkinsonism (Sibon and Tison, 2004; Thanvi et al., 2005; Lorberboym et al., 2006). Drug-induced downregulation of DaT expression is certainly a possibility. D_2_-receptor blockade may coexist with a dopamine nigrostriatal terminal defect, as assessed by DaT-SPECT abnormalities, in a relevant proportion of patients with drug induced parkinsonism (Schneider et al., 2007). In other words, DaT-SPECT imaging does not predict whether a given neuroleptic-treated patient will develop parkinsonism or not.

Functional or “psychogenic” parkinsonism is well recognized but relatively rare. If there is doubt about this diagnosis, careful follow up and functional imaging may resolve the uncertainty as studies are usually normal. However, contrary to what one would expect, several authors reported abnormal DaT-SPECT findings in a surprisingly high number of patients clinically diagnosed as having psychogenic (Lang et al., 1995; Hallett, 2011; Lang and Voon, 2011). It remains unknown whether these imaging changes are functional or structural in nature. In our study only 1 patient was diagnosed with psychogenic parkinsonian syndrome.

Multiple system atrophy (MSA), also known as striatonigral degeneration or one variant as Shy-Drager syndrome is characterized by a variable combination of parkinsonism, cerebellar ataxia and/or autonomic dysfunction (Ubhi et al., 2011; Ahmed et al., 2012). In our study 2 patients were diagnosed with MSA. They progressed quickly although responded well to L-DOPA initially.

Corticobasal degeneration is characterized by a combination of atypical parkinsonism and higher cortical dysfunction, even when one of these often dominates the clinical picture. Decreased presynaptic dopamine transporter binding have been found in most CBD patients (Hossain et al., 2003; Klaffke et al., 2006). Other study in a large CBS population found DaT-SPECT to be normal in about 10% of cases despite prominent bilateral extrapyramidal signs. In these cases, clinical and neuropsychological features were not distinct from those with evidence of SNc neuronal loss. The lack of any correlation between presynaptic nigrostriatal dysfunction and disease duration might suggest an unpredictable and possibly delayed SNc degeneration in CBD and further supports the hypothesis of a variable contribution of supranigral pathology to its motor phenotype (Cilia et al., 2011). In our series only 1 patient was diagnosed with corticobasal degeneration. She progressed toward dementia and severe dyspraxia quickly.

Repeating DaT-SPECT studies over time can reduce the diagnostic uncertainty that is present even after a prolonged period of observation (Vlaar et al., 2007; Antonini and Isaias, 2009). Of the 12 cases in which we repeated a second SPECT, the result changed to abnormal in 4. These patients were diagnosed with Parkinson's disease. However, after a second DaT-SPECT some patients still remain undiagnosed and other studies (such as PET studies) or even longer times of follow up are needed to move these patients in the diagnostic classification from “SWEDDs” to true nosological entities. As we have seen here, it seems clear that some patients with SWEDDs (those with predominant tremor subtype) have in fact dystonia, whereas others have other entities which are also well-known but may need some reassessment and follow-up to reach the diagnosis. In addition, it is conceivable that a number of patients might suffer from a disorder that has not yet been described.

Some patients with clinically uncertain parkinsonian syndrome exhibit outstanding frontal dysfunction. It is well known that cognitive impairment in early Parkinson's disease and other synucleinpathies (Parkinson-plus syndromes) are accompanied by reductions in activity in frontostriatal neural circuitry (Zgaljardic et al., 2003, 2004). These patients usually have an abnormal DaT-SPECT as the damage in frontostriatal neural circuitry occurs from down (basal ganglia) to up (frontal cortex) (Zgaljardic and Feigin, 2004). We speculate that some of the patients with clinically uncertain parkinsonian syndrome and normal DaT-SPECT studies may have a frontal neurodegenerative process involving fronto-subcortical circuits from top to bottom, that remains to be described. We have the hypothesis that some kind of neurodegenerative disorder affecting motor frontosubcortical circuits primary may exist, where parkinsonism is remarkable and frequently the first manifestation besides a disejecutive syndrome, although these patients do not usually meet the full criteria of frontotemporal lobe dementia. Functional neuroimaging and eventually neuropathological studies should be done in a cohort of these patients to confirm this extreme.

In conclusion, DaT-SPECT can be useful in the process of diagnosing tremor but it should not be relied on as a substitute for a careful, experienced clinical assessment and follow up. It is important to be well aware of the clinical clues to differentiate between these disorders. There is a list of alternative diagnoses to consider when a patient with tremor presents with normal DaT-SPECT, including disorders where it is expected to be normal, such essential tremor, dystonic tremor, vascular parkinsonism, drug induced parkinsonism among others, although the diagnosis of presynaptic parkinsonisms, including Parkinson's disease, is possible too. Repeating DaT-SPECTs over time may be useful in some circumstances since it can reduce the remaining diagnostic uncertainty that is present even after a prolonged period of observation. Especially, clinicians should consider repeating the DaT-SPECT when the clinical picture is consistent with typical early Parkinson's disease. However, after a second DaT-SPECT some patients still remain undiagnosed and other studies or even longer times of follow up are needed to move the diagnose of these patients from “SWEDDs” to true nosological entities.

## Author contributions

Manuel Menéndez-González, Francisco Tavares, Nahla Zeidan, José M. Salas-Pacheco, and Oscar Arias-Carrión designed, analyzed and performed research. All authors contributed to and have approved the final manuscript.

### Conflict of interest statement

The authors declare that the research was conducted in the absence of any commercial or financial relationships that could be construed as a potential conflict of interest.

## References

[B1] Ahmed Z. Asi Y. T. Sailer A. Lees A. J. Houlden H. Revesz T. (2012). The neuropathology, pathophysiology and genetics of multiple system atrophy. Neuropathol. Appl. Neurobiol. 38, 4–24 10.1111/j.1365-2990.2011.01234.x 22074330

[B2] Antonini A. Isaias I. U. (2009). Imaging evidence supports a link between essential tremor and Parkinson's disease. Nucl. Med. Commun. 30, 93–94 10.1097/MNM.0b013e328313e580 19194209

[B3] Apartis E. Blancher A. Meissner W. G. Guyant-Marechal L. Maltete D. De Broucker T. (2012). FXTAS: new insights and the need for revised diagnostic criteria. Neurology 79, 1898–1907 10.1212/WNL.0b013e318271f7ff 23077007

[B4] Asenbaum S. Pirker W. Angelberger P. Bencsits G. Pruckmayer M. Brucke T. (1998). [123I]beta-CIT and SPECT in essential tremor and Parkinson's disease. J. Neural Transm. 105, 1213–1228 9928890 10.1007/s007020050124

[B5] Bajaj N. P. Gontu V. Birchall J. Patterson J. Grosset D. G. Lees A. J. (2010). Accuracy of clinical diagnosis in tremulous parkinsonian patients: a blinded video study. J. Neurol. Neurosurg. Psychiatry 81, 1223–1228 10.1136/jnnp.2009.193391 20547625

[B6] Bajaj N. P. Wang L. Gontu V. Grosset D. G. Bain P. G. (2012). Accuracy of subjective and objective handwriting assessment for differentiating Parkinson's disease from tremulous subjects without evidence of dopaminergic deficits (SWEDDs): an FP-CIT-validated study. J. Neurol. 259, 2335–2340 10.1007/s00415-012-6495-5 22532169

[B7] Benitez-Rivero S. Marin-Oyaga V. A. Garcia-Solis D. Huertas-Fernandez I. Garcia-Gomez F. J. Jesus S. (2013). Clinical features and 123I-FP-CIT SPECT imaging in vascular parkinsonism and Parkinson's disease. J. Neurol. Neurosurg. Psychiatry 84, 122–129 10.1136/jnnp-2012-302618 22906618

[B8] Booij J. Speelman J. D. Horstink M. W. Wolters E. C. (2001). The clinical benefit of imaging striatal dopamine transporters with [123I]FP-CIT SPET in differentiating patients with presynaptic parkinsonism from those with other forms of parkinsonism. Eur. J. Nucl. Med. 28, 266–272 10.1007/s002590000460 11315592

[B9] Chabolla D. R. Maraganore D. M. Ahlskog J. E. O'Brien P. C. Rocca W. A. (1998). Drug-induced parkinsonism as a risk factor for Parkinson's disease: a historical cohort study in Olmsted County, Minnesota. Mayo Clin. Proc. 73, 724–727 10.4065/73.8.724 9703296

[B10] Chaudhuri K. R. Buxton-Thomas M. Dhawan V. Peng R. Meilak C. Brooks D. J. (2005). Long duration asymmetrical postural tremor is likely to predict development of Parkinson's disease and not essential tremor: clinical follow up study of 13 cases. J. Neurol. Neurosurg. Psychiatry 76, 115–117 10.1136/jnnp.2004.046292 15608009 PMC1739293

[B11] Cilia R. Rossi C. Frosini D. Volterrani D. Siri C. Pagni C. (2011). Dopamine transporter SPECT imaging in corticobasal syndrome. PLoS ONE 6:e18301 10.1371/journal.pone.0018301 21559307 PMC3085517

[B12] Cohen O. Pullman S. Jurewicz E. Watner D. Louis E. D. (2003). Rest tremor in patients with essential tremor: prevalence, clinical correlates, and electrophysiologic characteristics. Arch. Neurol. 60, 405–410 10.1001/archneur.60.3.405 12633153

[B13] Defazio G. Gigante A. F. Abbruzzese G. Bentivoglio A. R. Colosimo C. Esposito M. (2013). Tremor in primary adult-onset dystonia: prevalence and associated clinical features. J. Neurol. Neurosurg. Psychiatry 84, 404–408 10.1136/jnnp-2012-303782 23142961

[B14] De La Fuente-Fernandez R. (2012). Role of DaTSCAN and clinical diagnosis in Parkinson disease. Neurology 78, 696–701 10.1212/WNL.0b013e318248e520 22323748

[B15] Deuschl G. Bain P. Brin M. (1998). Consensus statement of the movement disorder society on tremor. Ad *hoc* Scientific Committee. Mov. Disord. 13(Suppl. 3), 2–23 10.1002/mds.870131303 9827589

[B16] Gerasimou G. Costa D. C. Papanastasiou E. Bostanjiopoulou S. Arnaoutoglou M. Moralidis E. (2012). SPECT study with I-123-Ioflupane (DaTSCAN) in patients with essential tremor. Is there any correlation with Parkinson's disease? Ann. Nucl. Med. 26, 337–344 10.1007/s12149-012-0577-4 22382608

[B17] Hallett M. (2011). Psychogenic parkinsonism. J. Neurol. Sci. 310, 163–165 10.1016/j.jns.2011.03.019 21458829 PMC3139799

[B18] Hossain A. K. Murata Y. Zhang L. Taura S. Saitoh Y. Mizusawa H. (2003). Brain perfusion SPECT in patients with corticobasal degeneration: analysis using statistical parametric mapping. Mov. Disord. 18, 697–703 10.1002/mds.10415 12784276

[B19] Huang C. Ravdin L. D. Nirenberg M. J. Piboolnurak P. Severt L. Maniscalco J. S. (2013). Neuroimaging markers of motor and nonmotor features of Parkinson's disease: an 18f fluorodeoxyglucose positron emission computed tomography study. Dement. Geriatr. Cogn. Disord. 35, 183–196 10.1159/000345987 23445555

[B20] Isaias I. U. Canesi M. Benti R. Gerundini P. Cilia R. Pezzoli G. (2008). Striatal dopamine transporter abnormalities in patients with essential tremor. Nucl. Med. Commun. 29, 349–353 10.1097/MNM.0b013e3282f4d307 18317299

[B21] Jacquemont S. Hagerman R. J. Leehey M. Grigsby J. Zhang L. Brunberg J. A. (2003). Fragile X premutation tremor/ataxia syndrome: molecular, clinical, and neuroimaging correlates. Am. J. Hum. Genet. 72, 869–878 10.1086/374321 12638084 PMC1180350

[B22] Klaffke S. Kuhn A. A. Plotkin M. Amthauer H. Harnack D. Felix R. (2006). Dopamine transporters, D2 receptors, and glucose metabolism in corticobasal degeneration. Mov. Disord. 21, 1724–1727 10.1002/mds.21004 16773621

[B23] Labiano-Fontcuberta A. Benito-Leon J. (2012). [Essential tremor and Parkinson's disease: are they associated?]. Rev. Neurol. 55, 479–489 23055430

[B24] Lang A. E. Koller W. C. Fahn S. (1995). Psychogenic parkinsonism. Arch. Neurol. 52, 802–810 10.1001/archneur.1995.00540320078015 7639632

[B25] Lang A. E. Voon V. (2011). Psychogenic movement disorders: past developments, current status, and future directions. Mov. Disord. 26, 1175–1186 10.1002/mds.23571 21626561

[B26] Lopez-Sendon J. Mena M. A. de Yébenes J. G. (2013). Drug-induced parkinsonism. Expert Opin. Drug Saf. 12, 487–496 10.1517/14740338.2013.787065 23540800

[B27] Lorberboym M. Treves T. A. Melamed E. Lampl Y. Hellmann M. Djaldetti R. (2006). [123I]-FP/CIT SPECT imaging for distinguishing drug-induced parkinsonism from Parkinson's disease. Mov. Disord. 21, 510–514 10.1002/mds.20748 16250023

[B28] Lorenzo Bosquet C. Miquel Rodriguez F. Roca Bielsa I. Mila M. Aguade Bruix S. Castell Conesa J. (2004). [Differential diagnosis of parkinsonism using dopamine transporters brain SPECT]. Med. Clin. (Barc.) 122, 325–328 10.1016/S0025-7753(04)74224-4 15033050

[B29] Marshall V. L. Reininger C. B. Marquardt M. Patterson J. Hadley D. M. Oertel W. H. (2009). Parkinson's disease is overdiagnosed clinically at baseline in diagnostically uncertain cases: a 3-year European multicenter study with repeat [123I]FP-CIT SPECT. Mov. Disord. 24, 500–508 10.1002/mds.22108 19117369

[B30] Nath U. Ben-Shlomo Y. Thomson R. G. Lees A. J. Burn D. J. (2003). Clinical features and natural history of progressive supranuclear palsy: a clinical cohort study. Neurology 60, 910–916 10.1212/01.WNL.0000052991.70149.68 12654952

[B31] Rajput A. Robinson C. A. Rajput A. H. (2004). Essential tremor course and disability: a clinicopathologic study of 20 cases. Neurology 62, 932–936 10.1212/01.WNL.0000115145.18830.1A 15037695

[B32] Rodriguez-Oroz M. C. Jahanshahi M. Krack P. Litvan I. Macias R. Bezard E. (2009). Initial clinical manifestations of Parkinson's disease: features and pathophysiological mechanisms. Lancet Neurol. 8, 1128–1139 10.1016/S1474-4422(09)70293-5 19909911

[B33] Schneider S. A. Edwards M. J. Mir P. Cordivari C. Hooker J. Dickson J. (2007). Patients with adult-onset dystonic tremor resembling parkinsonian tremor have scans without evidence of dopaminergic deficit (SWEDDs). Mov. Disord. 22, 2210–2215 10.1002/mds.21685 17712858

[B34] Schwingenschuh P. Ruge D. Edwards M. J. Terranova C. Katschnig P. Carrillo F. (2010). Distinguishing SWEDDs patients with asymmetric resting tremor from Parkinson's disease: a clinical and electrophysiological study. Mov. Disord. 25, 560–569 10.1002/mds.23019 20131394 PMC2996567

[B35] Serrano Vicente J. Garcia Bernardo L. Duran Barquero C. Constantino Silva A. Infante De La Torre J. R. Dominguez Grande M. L. (2009). [Negative predictive value of (123)I Ioflupane SPECT in movement disorders]. Rev. Esp. Med. Nucl. 28, 2–5 10.1016/S0212-6982(09)70207-1 19232169

[B36] Sibon I. Tison F. (2004). Vascular parkinsonism. Curr. Opin. Neurol. 17, 49–54 10.1097/00019052-200402000-00009 15090877

[B37] Stamelou M. De Silva R. Arias-Carrion O. Boura E. Hollerhage M. Oertel W. H. (2010). Rational therapeutic approaches to progressive supranuclear palsy. Brain 133, 1578–1590 10.1093/brain/awq115 20472654

[B38] Surasi D. S. Peller P. J. Szabo Z. Mercier G. Subramaniam R. M. (2013). Dopamine Transporter SPECT Imaging in Parkinson Disease and Dementia. PET Clin. 8, 459–467 10.1016/j.cpet.2013.08.006 27156473

[B39] Thanvi B. Lo N. Robinson T. (2005). Vascular parkinsonism–an important cause of parkinsonism in older people. Age Ageing 34, 114–119 10.1093/ageing/afi025 15713855

[B40] Tinazzi M. Cipriani A. Matinella A. Cannas A. Solla P. Nicoletti A. (2012). [(1)(2)(3)I]FP-CIT single photon emission computed tomography findings in drug-induced Parkinsonism. Schizophr. Res. 139, 40–45 10.1016/j.schres.2012.06.003 22727453

[B41] Tinazzi M. Fasano A. Di Matteo A. Conte A. Bove F. Bovi T. (2013). Temporal discrimination in patients with dystonia and tremor and patients with essential tremor. Neurology 80, 76–84 10.1212/WNL.0b013e31827b1a54 23243072

[B42] Tolosa E. Borght T. V. Moreno E. Da, TSCAN Clinically Uncertain Parkinsonian Syndromes Study Group (2007). Accuracy of DaTSCAN (123I-Ioflupane) SPECT in diagnosis of patients with clinically uncertain parkinsonism: 2-year follow-up of an open-label study. Mov. Disord. 22, 2346–2351 10.1002/mds.21710 17914722

[B43] Ubhi K. Low P. Masliah E. (2011). Multiple system atrophy: a clinical and neuropathological perspective. Trends Neurosci. 34, 581–590 10.1016/j.tins.2011.08.003 21962754 PMC3200496

[B44] Vlaar A. M. De Nijs T. Kessels A. G. Vreeling F. W. Winogrodzka A. Mess W. H. (2008). Diagnostic value of 123I-ioflupane and 123I-iodobenzamide SPECT scans in 248 patients with parkinsonian syndromes. Eur. Neurol. 59, 258–266 10.1159/000115640 18264015

[B45] Vlaar A. M. Van Kroonenburgh M. J. Kessels A. G. Weber W. E. (2007). Meta-analysis of the literature on diagnostic accuracy of SPECT in parkinsonian syndromes. BMC Neurol. 7:27 10.1186/1471-2377-7-27 17764571 PMC2064928

[B46] Williams D. R. De Silva R. Paviour D. C. Pittman A. Watt H. C. Kilford L. (2005). Characteristics of two distinct clinical phenotypes in pathologically proven progressive supranuclear palsy: Richardson's syndrome and PSP-parkinsonism. Brain 128, 1247–1258 10.1093/brain/awh488 15788542

[B47] Zgaljardic D. J. Borod J. C. Foldi N. S. Mattis P. (2003). A review of the cognitive and behavioral sequelae of Parkinson's disease: relationship to frontostriatal circuitry. Cogn. Behav. Neurol. 16, 193–210 14665819 10.1097/00146965-200312000-00001

[B48] Zgaljardic D. J. Feigin A. (2004). Neuroimaging of Parkinson's disease and atypical parkinsonism. Curr. Neurol. Neurosci. Rep. 4, 284–289 10.1007/s11910-004-0053-1 15217542

[B49] Zgaljardic D. J. Foldi N. S. Borod J. C. (2004). Cognitive and behavioral dysfunction in Parkinson's disease: neurochemical and clinicopathological contributions. J. Neural Transm. 111, 1287–1301 10.1007/s00702-004-0178-z 15480839

